# The contributions of the tissue inhibitor of metalloproteinase-1 genotypes to triple negative breast cancer risk

**DOI:** 10.7603/s40681-016-0004-6

**Published:** 2016-02-23

**Authors:** Wen-Shin Chang, Liang-Chih Liu, Chieh-Lun Hsiao, Chen-Hsien Su, Hwei-Chung Wang, Hong-Xue Ji, Chia-Wen Tsai, Ming-Chei Maa, Da-Tian Bau

**Affiliations:** 1 Terry Fox Cancer Research Laboratory, China Medical University Hospital, 404 Taichung, Taiwan; 2 Graduate Institute of Clinical Medical Science, China Medical University, 404 Taichung, Taiwan; 3 Graduate Institute of Basic Medical Science, China Medical University, 404 Taichung, Taiwan; 4 Department of Bioinformatics and Medical Engineering, Asia University, 413 Taichung, Taiwan; 5 Terry Fox Cancer Research Laboratory, Department of Medical Research, China Medical University Hospital, 404 No. 2, Yuh-Der Road, Taichung, Taiwan

**Keywords:** Breast cancer, Genotype, MMP, Polymorphism, Taiwan, TIMP-1, Triple negative breast cancer

## Abstract

The tissue inhibitors of metalloproteinases (TIMPs) are a family of multifunctional proteins which have been shown to be upregulated in various types of cancers. However, the contribution of TIMPs in breast cancer is not fully understood, not to mention triple negative breast cancer (TNBC). This study’s aim was to evaluate the contribution of *TIMP-1* rs4898, rs6609533, and rs2070584 genotypes to the risk of breast cancer, especially the subtype of TNBC. The contributions of these *TIMP-1* genotypes to cancer risk were examined among 1232 breast cancer patients and 1232 healthy controls, and several clinicopathologic factors were also analyzed. The results showed that the percentages of CC, CT, and TT of *TIMP-1* rs4898 were differentially distributed at 28.5%, 33.1% and 38.4% in the breast cancer patient group and 34.5%, 41.0% and 24.5% in the control group, respectively (*P* for trend = 7.99*10^-13^). It was also found that the CC genotype carriers were of increased risk for breast cancer (odds ratio = 1.90, 95% confidence interval = 1.55-2.33, *P* = 0.0001) than the TT genotype carriers. In addition, we analyzed the allelic frequency distributions of all three *TIMP-1*s, and the results showed that the C allele of *TIMP-1* rs4898 contributes to an increase in breast cancer susceptibility (*P* = 2.41*10^-12^). On the other hand, there was no difference found in the distribution of genotypic or allelic frequencies among the patients and the controls for *TIMP-1* rs6609533 and rs2070584. Thus, it is our conclusion that the CC genotype of *TIMP-1* rs4898 compared to the TT wild-type genotype may increase the risk for breast cancer, especially TNBC in Taiwan, and may serve as an early detective and predictive marker.

## 1. Introduction

Statistically, breast cancer is one of the most common malignancies all over the world, and triple negative breast cancer (TNBC) is the most invasive form, with a poor prognosis and a high percentage chance to recur locally and metastasize distantly [1]. At present, no standard therapy has been clinically feasible for TNBC, and the discovery of useful biomarkers is urgently needed. In Taiwan, breast cancer ranks second among the common types of cancer, and has been noted for its high incidence, high mortality, and early onset [2, 3].

In the early 1990s, scientists knew that the dysregulation of the extracellular matrix (ECM) contributes to the initial phase of micro-environmental remodeling during physiological processes such as morphogenesis, angiogenesis, inflammation, wound healing, and tumorigenesis [4]. Among the various kinds of ECM component molecules, the matrix metalloproteinases (MMPs) are a group of endopeptidases that play a key role in ECM remodeling [5], and MMPs have been reported to have the capacity to degrade the connective tissue matrices [4, 5]. Meanwhile, each MMP is under the control of their specific inhibitors, e.g., the tissue inhibitors of metalloproteinases (TIMPs) [5]. For instance, MMP-1 and MMP-2 are in charge of the degradation of the connective tissue fibrillary collagen (type I collagen) and type IV globular basement membrane collagen, respectively [4]. The expressions of MMP-1 and MMP-2 are under the control of their specific inhibitors TIMP-1 and TIMP-2, respectively [5]. An imbalance of MMPs and TIMPs results in metalloproteinase activation, and relatively higher levels of MMPs than TIMPs may stimulate the degradation of collagen in the interstitial space and beneath epithelial and endothelial cells, leading to acute injury [6].

**Table 1:** 

Characteristics	Controls (n = 1232)			Patients (n = 1232)			*P*-value
	n	%	Mean (SD)	n	%	Mean (SD)	
Age (years)							
< 40	359	29.1%		362	29.4%		0.89^a^
40-55	558	45.3%		547	44.4%		
> 55	315	25.6%		323	26.2%		
Age at menarche (years)			12.4 (0.7)			12.1 (0.6)	0.79^b^
Age at birth of first child (years)			29.4 (1.2)			29.8 (1.4)	0.63^b^
Age at menopause (years)			48.8 (1.8)			49.3 (2.0)	0.59^b^
Site							
Unilateral				1198	97.2%		
Bilateral				34	2.8%		
Family History							
First degree (Mother, sister and daughter)				55	4.5%		
Second degree				6	0.5%		
No history				1171	95%		
Habits							
Cigarette smokers	86	7.0%		170	13.8%		< 0.0001^a^
Alcohol drinkers	91	7.4%		162	13.1%		< 0.0001^a^

Statistical results based on ^a^ *Chi*-square or ^b^unpaired *Student’s t*-test.

Up to now, the genomic contribution of *TIMP-1* to cancer has not been well elucidated. As for breast cancer, the *TIMP-1* genotype has not been found to contribute to breast cancer risk yet. However, the genotype of *TIMP-2* has [7]. In that study, the variant CT and TT genotypes at the polymorphic site C-1306T of *TIMP-2* (rs243865) were associated with a reduced risk of breast cancer (OR = 0.490, 95% CI = 0.033-0.730), compared with the wild-type CC genotype, among 210 breast cancer patients and 290 healthy Tunisian women [7]. Now, in the current study, we are going to evaluate whether the genotypes of *TIMP-1* are associated with an individual’s susceptibility to breast cancer, and whether these polymorphisms are associated with more invasive and aggressive TNBC.

## 2. Materials and methods

### 2.1. Investigated population

One thousand two hundred and thirty two patients diagnosed with breast cancer were recruited from the outpatient clinics of general surgery at the China Medical University Hospital in Taiwan for this study. The clinical characteristics of the patients, including histological details, were all defined by the expert surgeons Dr. Wang, Dr. Liu, and their teammates. The slides of the cancer tissues were reviewed and scored by at least two independent pathologists. For ER, PR, and p53 immunoassaying, nuclear staining in 10% of neoplastic cells was used as a positive cutoff. A Ki67-labelling index of > 30% was considered positive. HER-2/neu results were determined according to the package insert and guidelines of the American Society of Clinical Oncology and the College of American Pathologists [8]. All patients voluntarily participated, completed a self-administered questionnaire and provided peripheral blood samples. 1232 age-matched healthy volunteers were selected after an initial random sampling from the Health Examination Cohort of the hospital as the controls of this study. The exclusion criteria of the control group included previous malignancy, metastasized cancer from other or unknown origin, and any familial or genetic diseases. Both groups completed a short questionnaire which included habits. Our study was approved by the Institutional Review Board of the China Medical University Hospital (DMR96-IRB-240) and written informed consent was obtained from all participants.

### 2.2. Genotyping conditions

Genomic DNA from the peripheral blood leucocytes of each recruited subject was prepared using the QIAamp Blood Mini Kit (Blossom, Taipei, Taiwan) and further processed as in our previous articles [9, 10]. The polymerase chain reaction (PCR) cycling conditions were as follows: one cycle at 94°C for 5 min; 35 cycles at 94°C for 30 s, 55°C for 30 s, and 72°C for 30 s; and a final extension at 72°C for 10 min. The sequences of forward and reverse primers and the restriction enzymes for each SNP were designed by our lab and are summarized in Table 2. Each genotypic procession was performed by two translational researchers independently and blindly. Five percent of the total samples for rs6609533 were randomly selected for direct sequencing, and the results from PCR-RFLP and direct sequencing were 100% concordant. The results from direct sequencing for rs4898 and rs2070584 were also 100% concordant between the results using forward and reverse primers.

### 2.3. Statistical analyses and significance identification

All of the 1232 controls and 1232 cases with genotypic and clinical data were analyzed. To ensure that the controls used were representative of the general population and to exclude the possibility of any genotyping errors, the deviation of the genotype frequencies of *TIMP-1* SNPs in the control subjects from those expected under the Hardy-Weinberg equilibrium was assessed using the goodness-of-fit test. Pearson’s Chi-square test was used to compare the distribution of the *TIMP-1* genotypes between case subjects and control subjects. The associations between the *TIMP-1* polymorphisms and breast cancer risk were estimated by calculating odds ratios (ORs), as well as their 95% confidence intervals (CIs) from logistic regression analysis. Any *P* < 0.05 was considered statistically significant, and all statistical tests were performed with a two-side examination.

**Table 2:** 

Polymorphisms (locations)	Primer sequences	Restriction enzyme	SNP sequence	DNA fragment size (bp)
rs4898	F: 5’-TTCAGTCTATCAGAAGGCCG-3’ R: 5’-TAGCAAGAGAGATCAGGGAC-3’		T C	Direct sequencing
rs6609533	F: 5’-CCTCTGGCTATTCTGTGTCC-3’ R: 5’-AGAGCAACAAGCAGATGTGC-3’	*Bcl* I	G A	312 bp 175 + 137 bp
rs2070584	F: 5’-GTTGCTGATGACCTGGTGTG-3’ R: 5’-TGAGGATGAGGACAGTAACA-3’		T G	Direct sequencing

*F and R indicate forward and reverse primers, respectively.

**Table 3:** 

Genotypes	Controls		Patients		OR (95% CI)	*P*-value^a^
	n	%	n	%		
rs4898						
TT	425	34.5%	351	28.5%	1.00 (reference)	
CT	505	41.0%	407	33.1%	0.98 (0.81-1.18)	0.8063
CC	302	24.5%	474	38.4%	1.90 (1.55-2.33)*	0.0001*
*P*_trend_						**7.99 × 10** ^-13*^
rs6609533						
AA	488	39.6%	479	38.9%	1.00 (reference)	
AG	373	30.3%	398	32.3%	1.09 (0.90-1.31)	0.4118
GG	371	30.1%	355	28.8%	0.97 (0.80-1.18)	0.8061
*P*_trend_						0.5361
rs2070584						
TT	503	40.8%	488	39.6%	1.00 (reference)	
GT	406	33.0%	433	35.1%	1.10 (0.91-1.32)	0.3246
GG	323	26.2%	311	25.3%	0.99 (0.81-1.21)	0.9595
*P*_trend_						0.5161

^a^Based on *Chi*-square test; *P < 0.05.

## 3. Results

A total of 1232 patients diagnosed with breast cancer and the same amount of matched controls were included in this study, and their data are compared and summarized in Table 1. The ages of patients and controls are all well matched and no difference in age, age at menarche, and age at birth of first child among the subjects were observed (*P* = 0.05) (Table 1). As for individual behaviors, cigarette smoking and alcoholism were both risk factors for breast cancer in this population (*P* < 0.05) (Table 1).

The distributions of the *TIMP-1* genotypes at rs4898, rs6609533, and rs2070584 among the non-cancer controls and the breast cancer patients are presented and statistically analyzed in Table 3. In the top of Table 3, the results show that the genotypes of *TIMP-1* rs4898 were differentially distributed between breast cancer and control groups (*P* for trend = 7.99*10^-13^) (Table 3). In detail, the *TIMP-1* rs4898 heterozygous CT was not associated with breast cancer risk (OR = 0.98, 95% CI = 0.81-1.18, *P* = 0.8063), while the homozygous CC genotype seemed to be associated with increased breast cancer risk (OR = 1.90, 95% CI = 1.55-2.33) (Table 3). On the other hand, there was no association between the genotype of either rs6609533 or rs2070584 and breast cancer risk (Table 3).

In order to confirm the findings in Table 3, the analysis of allelic frequency distribution for the three *TIMP-1* SNPs was also performed, and the results are summarized in Table 4. Consistent with the findings that the homozygous CC genotype of *TIMP-1* rs4898 is associated with increased breast cancer risk, the allele C was 55.0% in the patient group, significantly higher than that of 45.0% in the control group (*P* = 2.41*10^-12^). Again, there was no significant difference in the distribution of allelic frequencies for rs6609533 or rs2070584 between the control and breast cancer patient groups (Table 4).

**Table 4:** 

Alleles	Controls	%	Patients	%	*P*-value^a^
rs4898					
Allele T	1355	55.0%	1109	45.0%	**2.41 × 10** ^-12*^
Allele C	1109	45.0%	1355	55.0%	
rs6609533					
Allele G	1349	54.7%	1356	55.0%	0.8412
Allele A	1115	45.3%	1108	45.0%	
rs2070584					
Allele T	1412	57.3%	1409	57.2%	0.9312
Allele G	1052	42.7%	1055	42.8%	

^a^ *P*-value based on *Chi*-square test.

*Statistically identified as significant.

**Table 5:** 

Characters	Genotype, number (%)^a^			OR (95% CI)^b^	*P*-value^c^
	TT	CT	CC		
Control	425 (34.5)	505 (41.0)	302 (24.5)	1.00 (Ref.d)	
Triple-negative status					
No	166 (30.0)	238 (43.0)	149 (27.0)	1.23 (0.99-1.52)	0.1662
Yes	24 (23.1)	34 (32.7)	46 (44.2)	1.76 (1.10-2.81)*	**5.35 × 10** ^-5^*
Ki67 status					
Negative	101 (36.5)	104 (37.5)	72 (25.0)	0.92 (0.70-1.20)	0.5725
Positive	122 (36.1)	116 (34.3)	100 (29.6)	0.93 (0.73-1.20)	0.0538

^a^ Triple-negative and Ki67 status data were available for 657 and 615 patients, respectively. All data are given as number of patients (%) unless otherwise noted.

^b^ OR, odds ratio; CI, confidence interval, variant CT + CC versus TT.

^c^ Based on 3*2 Chi-square test.

^d^ Ref., reference.

*Statistically significant

We are interested in the association of clinicopathologic characteristics with *TIMP-1* rs4898 genotypes. Given that distinct subtypes of breast cancer have different mechanisms of carcinogenesis, we analyzed the associations of *TIMP-1* rs4898 genotypes with the age-related and the clinicopathologic characteristics of breast cancer patients, and the results showed no differential distribution of the genotype at *TIMP-1* rs4898 among women who were younger than 55 years or older. Similarly, there was no differential distribution of the genotype at *TIMP-1* rs4898 among women with their first menarche earlier or later than 12.2 years, or with menopause earlier or later than 49.0 years (data not shown). In addition, the difference in the distribution of the genotype among breast cancer patients stratified by other factors, including first full pregnancy (data not shown) or Ki67 status (Table 5), was not statistically significant. The finding that should be highlighted is whether the *TIMP-1* rs4898 genotype was differentially distributed among those patients who were triple negative or not (*P* = 5.35*10^-5^) (Table 5). Another key finding is that the variant genotypes (CT and CC) contribute to an increased risk for TNBC (OR = 1.76, 95% CI = 1.10-2.81).

## 4. Discussion

In the present case-control study, the contributions of three *TIMP-1* SNPs, rs4898, rs6609533 and rs2070584 to the risk of breast cancer was firstly evaluated in a population in Taiwan. The highlight is that as for the rs4898, the CC genotype was significantly associated with an increased risk of breast cancer (Table 3). However, the heterogeneous CT genotype was not associated with a risk of breast cancer (Table 3). As for the other two SNPs, no obvious differential distribution in the genotypes of *TIMP-1* rs6609533 or *TIMP-1* rs2070584 was found (Table 3). The allelic frequency analysis suggests that the C allele of *TIMP-1* rs4898 is associated with an increased risk of breast cancer (4).

It is well known that the MMPs are in charge of the degradation of the basement membrane and the extracellular matrix. In normal conditions, MMPs are expressed at a relatively low level, and TIMP-1 proteins can bind with MMP-1 to suppress the activity of MMP-1 [11, 12]. The dynamic balance between MMPs and TIMPs plays a pivotal role in the homeostasis of normal conditions for our cells. Studies for the genotyping of *MMPs* seem to be much advanced than those for *TIMPs*. For example, in 2012 Liu and his colleagues performed a meta-analysis exploring the association between *MMP1* promoter -1607 1G/2G polymorphism and the risk of several types of cancer, and the results showed that an elevated risk of cancer was found regarding breast cancer and other types of cancer such as colorectal cancer and genitourinary neoplasm [13]. Thus, there is an urgent need for scientists to reveal the contributions of genotypes of other MMPs and TIMPs to common cancers. Also, combined analyses of *MMP-1* and *TIMP-1* genotypes may provide further data to evaluate the contribution of said genotypes to each single gene.

In recent years, the Terry Fox Cancer Research group in the China Medical University and Hospital has been continuously devoted to the genotyping work of breast cancer in Taiwan, and their efforts have provided some potential predictive biomarkers such as G-1394T (rs 6869366) in *XRCC4* [14], K589E (rs1047840) in *EXO1* [15], G-1401T (rs828907) in *XRCC5* [16], Asp312Asn (rs1799793) in *XPD* [17], rs189037 in *ATM* [18], G14713A and T29107A in *CAV-1* [19], C-802G (rs14133) in *CRYAB* [20], C677T (rs1801133) in *MTHFR* [21], and G-765C (rs20417) in *COX-2* [10]. The systematic analyses of clinicopathologic statuses have extended our understanding of the contributions of genotypes to TNBC [9, 22]. It goes without saying that it is very meaningful to identify those potential predictive markers for breast cancer and their subtypes, such as TNBC.

In conclusion, the current study provides evidence that the C allele of *TIMP-1* rs4898 contributes to an increased breast cancer risk, especially for those afflicted with TNBC. These findings may be helpful to the revealing of the genomic contributions of *TIMP-1* in other populations. The results of this study ought to be tested and either confirmed or denied in multi-center and multipopulation studies in the future.
